# Exploring the lived experience of renal cachexia for individuals with end-stage renal disease and the interrelated experience of their carers: Study protocol

**DOI:** 10.1371/journal.pone.0277241

**Published:** 2022-11-03

**Authors:** Carolyn Blair, Joanne Shields, Robert Mullan, William Johnston, Andrew Davenport, Denis Fouque, Kamyar Kalantar-Zadeh, Peter Maxwell, Clare McKeaveney, Helen Noble, Sam Porter, David Seres, Adrian Slee, Ian Swaine, Miles Witham, Joanne Reid

**Affiliations:** 1 School of Nursing and Midwifery, Queen’s University Belfast, Belfast, United Kingdom; 2 Regional Nephrology Unit, Belfast City Hospital, Belfast Health & Social Care Trust, Belfast, United Kingdom; 3 Renal Unit, Antrim Area Hospital, Northern Health & Social Care Trust, Antrim, United Kingdom; 4 Northern Ireland Kidney Patients Association, Belfast, United Kingdom; 5 UCL Department of Renal Medicine Royal Free Hospital University College London, London, United Kingdom; 6 Division of Nephrology, Dialysis and Nutrition, Hôpital Lyon Sud and University of Lyon, Pierre-Bénite, France; 7 Irvine Division of Nephrology, Hypertension and Kidney Transplantation, University of California, Irvine, CA, United States of America; 8 Centre for Public Health, Queen’s University Belfast, Belfast, United Kingdom; 9 Department of Social Sciences and Social Work, Bournemouth University, Poole, United Kingdom; 10 Institute of Human Nutrition and Department of Medicine, Columbia University Irving Medical Center, New York, NY, United States of America; 11 Faculty of Medical Sciences, University College London, London, United Kingdom; 12 School of Human Sciences, University of Greenwich, Greenwich, United Kingdom; 13 Campus for Ageing and Vitality, Newcastle University, Newcastle Upon Tyne, United Kingdom; Faculty of Medicine, Saint-Joseph University, LEBANON

## Abstract

Renal cachexia is an important consideration in the person-centred care that is needed in end-stage renal disease (ESRD). However, given that clinical guidelines relating to renal cachexia are largely absent, this is an unmet care need. To inform guidelines and future renal service planning, there is an urgency to understand individuals’ experiences of renal cachexia and the interrelated experiences of the carers in their lives. We report here the protocol for an interpretative phenomenological study which will explore this lived experience. A purposive sampling strategy will recruit individuals living with ESRD who have cachexia and their carers. A maximum of 30 participants (15 per group) dependent on saturation will be recruited across two nephrology directorates, within two healthcare trusts in the United Kingdom. Individuals with renal cachexia undergoing haemodialysis will be recruited via clinical gatekeepers and their carers will subsequently be invited to participate in the study. Participants will be offered the opportunity to have a face-to-face, virtual or telephone interview. Interviews will be audio-recorded, transcribed verbatim and analysed using interpretative phenomenological analysis. NVivo, will be used for data management. Ethical approval for this study was granted by the Office for Research Ethics Committees Northern Ireland (REC Reference: 22/NI/0107). Scientific evidence tends to focus on measurable psychological, social and quality of life outcomes but there is limited research providing in-depth meaning and understanding of the views of individuals with renal disease who are experiencing renal cachexia. This information is urgently needed to better prepare healthcare providers and in turn support individuals with ESRD and their carers. This study will help healthcare providers understand what challenges individuals with ESRD, and their carers face in relation to cachexia and aims to inform future clinical practice guidelines and develop supportive interventions which recognise and respond to the needs of this population.

## Introduction

Cachexia is a complex metabolic syndrome associated with various diseases [1] which includes ‘objective’ manifestations such as inadequate food intake, weight loss, inactivity, loss of muscle mass and metabolic derangements, inducing catabolism [2–4] and ‘subjective’ symptoms such as anorexia, early satiety, taste alterations, chronic nausea, distress, fatigue and loss of concentration [4]. In end-stage renal disease (ESRD), this syndrome is poorly defined and known as ‘renal cachexia’ [5, 6]. Currently there is no standardised definition or inclusion criteria for renal cachexia [7, 8]. Individuals with ESRD typically present with anorexia, muscular weakness and protein-energy wasting (PEW) [5, 9], the highest prevalence of wasting (estimated as up to 75%) [5] is among those receiving haemodialysis treatment and is associated with poor clinical outcomes [10, 11]. Renal cachexia is the most severe stage of PEW, and many individuals with PEW will eventually reach this stage [5, 6]. Intensive dietary support and dialysis can reverse deterioration in nutritional status for individuals with ESRD who have anorexia-induced insufficient energy intake [6, 10, 12, 13]. However, it is much more difficult to improve the nutritional status and body composition in individuals who have renal cachexia because of additional, profound metabolic alterations [6, 10, 12, 13]. Evidence about appropriate interventions is sparce and the need for robust studies to establish the effectiveness of potential treatments on renal cachexia has been identified [8, 14–16]. There is also very little known about the lived experience of renal cachexia [5, 14–17]. There is therefore an urgent need for Health Care Professionals (HCPs) to better understand the impact of this syndrome, in order to provide better care delivery.

In the first mixed methods study eliciting the views of HCPs about renal cachexia, McKeaveney et al., [14] reported that HCPs recognised that a lack of standards of care or guidelines for the treatment of renal cachexia made current practice highly variable. It was acknowledged by HCPs that “information material [relating to renal cachexia] is scarce or absent” and this lack of information can mean that individuals with ESRD are “very frightened” about changes in appetite that may be correlated with cachexia [14]. It is evident that food and eating are fundamental aspects of quality of life (QoL) which are impacted by cachexia in ESRD [14]. However, this remains an underdiagnosed and undertreated condition which has a devastating impact on QoL evident in other disease trajectories [18–22]. Findings in the study by McKeaveney et al. [14] suggest that HCPs deemed the most important factors when treating renal cachexia to be an improvement in QoL (69%) and the relief of family distress (41%). HCPs endeavour to deliver person-centred care, however to do so effectively, they need to understand the subjective and social processes involved, as articulated by individuals experiencing the condition, and their carers [7, 14, 23]. Evidence-based practice is founded on person-centred care and is promoted by The European Kidney Health Alliance [24], The UK Kidney Association Kidney [25] and Kidney Care UK [26] and aligns with the UK’s ‘National Service Framework for Renal Services’ [27] ‘Standard one: A patient-centred service’ to improve quality of life for individuals with renal disease and their carers. Symptom identification, amelioration and management of the condition are high priorities for individuals with renal disease, their carers and HCPs. Therefore, there is an urgent need for studies that could help to interpret these experiences, and thereby could inform Clinical Practice Guidelines (CPGs).

This study has been designed in response to the urgent need identified in the National Service Framework to provide evidence concerning individuals’ experiences with ESRD to inform clinical practice guidelines for management of renal cachexia. It will focus on the qualitative exploration of the views of those who are living with renal cachexia and the impact that this syndrome has on the lives of these individuals and their carers. Multi-perspective designs, such as this study involving carers, are necessary given that it is increasingly recognised that the experience of living with chronic disease “is not solely located within the accounts of those with the diagnosis” [28] (p.182). Although carers are not always included in research relating to individuals with ESRD, a systematic review relating to ESRD [29] suggests that further research is required to understand family members’ perspectives. Furthermore, including carers in research rightly acknowledges that this dyad is valued as an essential component in palliative care and is important when considering the future delivery of renal services. Therefore, the purpose of this study is to explore individuals’ rich qualitative perspectives of living with renal cachexia, with the aim of promoting crucial discussions about the implications that these perspectives might have for practice, including their views about potential targeted interventions to improve QoL. Here we present a protocol for this exploratory study.

## Materials and methods

### Research aim

To explore the lived experience of renal cachexia for individuals with ESRD and the interrelated experiences of their carers.

### Operational definition of ‘carer’ in this study

For the purposes of this study, the carer is a lay individual (i.e., non-HCP) who is seen as integral to the supportive journey for the individual living with renal cachexia, has regular (>5 times per week) face-to-face contact with them and is identified by the individual as their carer.

### Design

This exploratory, interpretative phenomenological study using semi-structured interviews has been designed using the consolidated criteria for reporting qualitative research (COREQ) [30].

### Setting

The study will be conducted within the nephrology directorate at Belfast Health and Social Care Trust (BHSCT) and the Northern Health and Social Care Trust (NHSCT).

#### Inclusion and exclusion criteria

The inclusion and exclusion criteria for participants are defined in Table 1. Any individual who displayed secondary causes of cachexia, where their weight loss resulted from a clinically explainable reduced oral intake rather than the metabolic processes associated with cachexia, will be excluded from this study. Excluding individuals with ESRD on these grounds reflects current research, which acknowledges that weight loss in cachexia is caused by more than just a reduction in food intake [4]. In reference to the criterion ‘Haemoglobin (<12 g/dl)’, anaemia is considered a feature of cachexia in chronic illness [1] and is an important marker which helps to distinguish between cachexia, sarcopenia and protein energy wasting in ESRD Patients [31] Anaemia is a frequent problem in patients with ESRD [31–33], according to large-scale population studies the incidence of anaemia (haemoglobin <12 g/dL) exceeds 70% in patients with ESRD [34–36], other studies indicate that in the dialysis population anaemia incidence is as high as 90% [37, 38]. In reference to the inclusion criteria for carers, we recognise that all informal carers who are identified as significant others do not always live with those with chronic illness therefore, we purposefully added that they must have ‘face to face’ contact more than 5 times per week. Our purpose in adding this criterion is to ensure that informal carers who do not live with the individual with renal cachexia are not excluded from the study.

**Table 1:** Inclusion criteria.

**Renal Population**	
**Inclusion Criteria**	**Exclusion Criteria**
Have a confirmed diagnosis of end-stage renal disease and in receipt of haemodialysis	Under 18 years of age
	Are living/nursed as an inpatient in hospital or a resident in a care home.

	Haemoglobin (>12 g/dl)
To be included in the study, participants will have: oedema-free weight loss of at least 5% in 12 months or less. In cases where weight loss is not documented a BMI <20.0 kg/m2 is sufficient; plus 3 of the following • fatigue—defined as physical and/or mental weariness resulting from exertion; an inability to continue exercise at the same intensity with a resultant deterioration in performance; • anorexia—limited food intake (i.e. total caloric intake less than 20 kcal/kg body weight/d, <70% of usual food intake) or poor appetite; • abnormal biochemistry: • increased inflammatory markers (CRP>5.0 mg/l), IL-6 >4.0 pg/ml) • low serum albumin (<3.2 g/dl) [1]). Criteria will be confirmed through the clinical gatekeeper.	Experiencing weight loss due to other physical causes. Any participant who displays secondary causes of renal cachexia will be excluded for example: malabsorption (i.e prolonged nausea or vomiting; oesophageal blockage; bowel obstruction; persistent diarrhoea), starvation, primary depression, hyperthyroidism and age-related loss of muscle mass. Criteria will be confirmed through the clinical gatekeeper.
Be alert and mentally competent (as assessed by a member of the multi-disciplinary team)	Cognitive impairment (as assessed by their consultant or health care team)
Have the ability to provide informed consent, read and write English	Non-English speaking.
Able/willing to be involved.	
**Informal Carer**	
**Inclusion Criteria**	**Exclusion Criteria**
Identified by the individual in the renal population as their significant other and has face-to-face contact with the individual in the renal population more than 5 times per week	Under 18 years of age
Alert and mentally competent (self-assessment)	Cognitive impairment (as assessed by their consultant or health care team)
Have the ability to provide informed consent, read and write English	Non-English speaking
Able/willing to be involved.	

### Sampling

Purposive sampling is a non-probability technique used to identify and choose information-rich cases for the most suitable use of available resources [39]. Selecting research participants using this method, who have lived experience of renal cachexia and those who have a close relationship with individuals living through this experience, will offer an in-depth understanding of the phenomenon. The use of purposive sampling is also congruent with the philosophy of interpretative phenomenological analysis, as those with lived experience of renal cachexia will willingly volunteer to share their lived experience [40]. Only participants who meet the stipulated inclusion criteria (Table 1) will be approached to take part in this study and only carers of those individuals who take part in the research will be approached. [41]. Therefore, it is possible for an individual from the renal population to be included in the study without a carer, however the reverse will not be the case. This specific population has been selected to provide depth of insight into the lived experience of cachexia is in ESRD and particularly among those receiving haemodialysis treatment [5, 10]. Sampling will be driven by the aspiration of learning in detail about the depth of experience of the research participants. Thus, the final sample size will be determined by the research team when it is agreed data saturation has been achieved [42].

### Recruitment procedure

**Fig 1:**
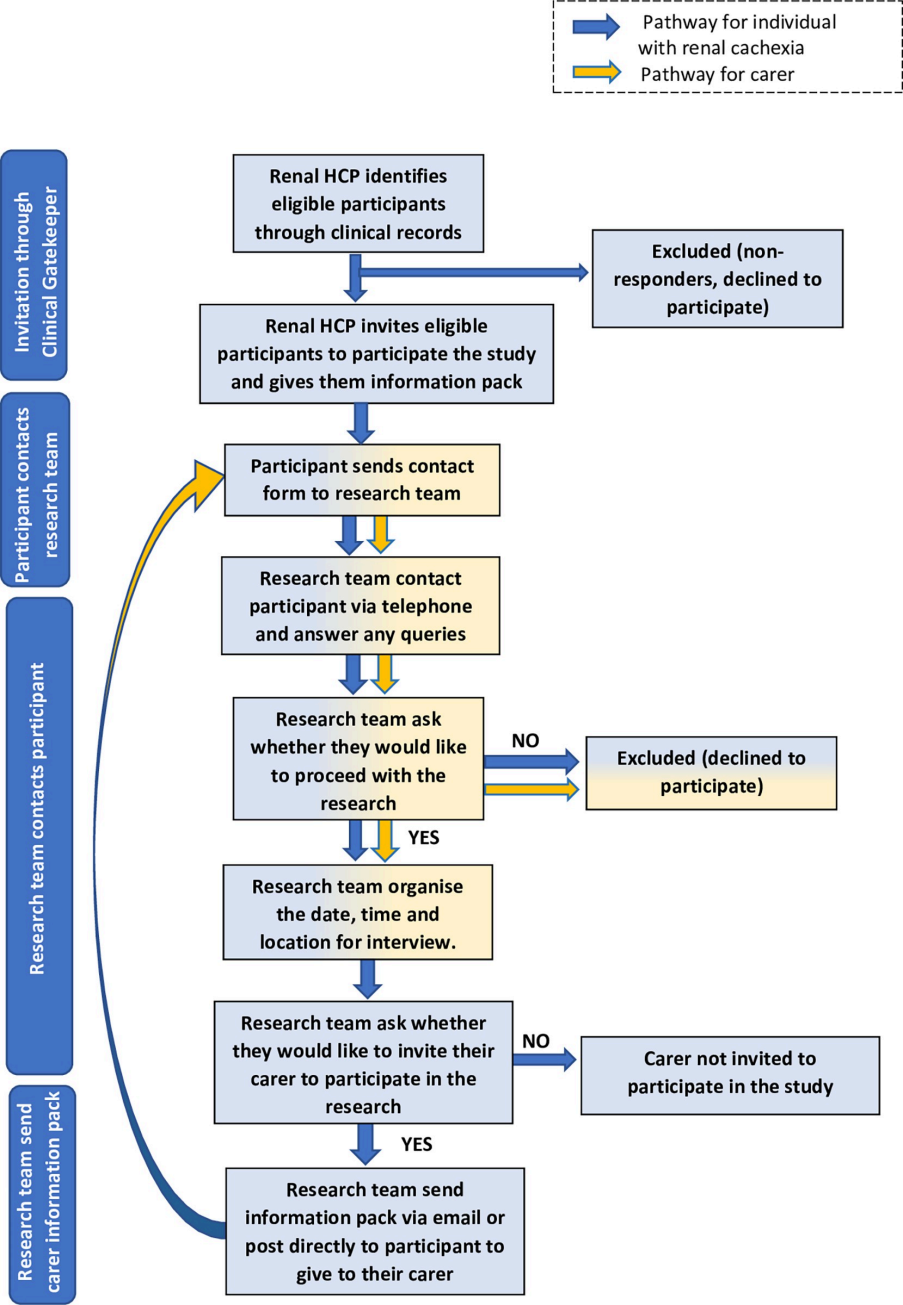
Recruitment flow chart.

The recruitment procedure will be promoted through clinical gatekeepers (consultant nephrologists) in two healthcare trusts within the United Kingdom. A maximum of 30 participants, in total (15 individuals with renal cachexia and 15 carers) will be recruited across the two trusts. Initially, approximately, 15 individuals with renal cachexia will be identified and invited by a member of the clinical care team to participate when attending routine haemodialysis appointments. Individuals with renal cachexia will be provided with an information pack, this will include an invitation letter, participant information sheet (PIS), and contact form which includes a request for contact details. If these individuals decide to participate, they will complete their contact form and return via email, to a member of the research team. Upon receipt of the contact form, a member of the research team will make a follow-up telephone call to answer any queries and discuss the interview process. On confirmation of involvement in the study the researcher will contact the clinical gatekeeper via phone and ask them to complete the screening proforma which is based on the inclusion criteria and Evan’s et al.’s [1] definition, (S1 File: Proforma for consenting participants). This method will ensure that each individual with ESRD who is interested in participating is eligible for the study. If the participant remains willing to take part, a convenient time, place and date for the interview will be scheduled. During this follow-up telephone call, a member of the research team will also ask these participants (who have agreed to participate) if they would like to invite their carer to participate in the study. Considering this study is predominantly focused on the experiences of those with renal cachexia from the perspective of the individual themselves and their informal carers, we deemed it necessary to maintain the dignity and privacy of these individuals though giving individuals with renal cachexia the choice as to whether they would like to nominate their informal carer for the study. However, informed consent will be gained directly from the informal carers in relation to their inclusion within the study. If in agreement, an information pack including the PIS for carers and contact form will be sent to individuals with renal cachexia via email (or post if preferred) to give to their carer. The process regarding contact and scheduling interviews will then be replicated with this population (Fig 1) aligned with the inclusion criteria for informal carers. Given that it is not possible for a carer to be involved in the study if the relevant individual with renal cachexia does not consent, we are aware that it is likely that we will have a lesser number of carers than individuals with renal cachexia involved in the study.

### Data collection

The researcher (CB) who is an experienced qualitative researcher, will be solely responsible for data collection to ensure consistency. One semi-structured interview will be conducted per participant, the suitability of this data collection technique in phenomenological research is delineated in the literature [40]. Participants will be offered either a face-to-face interview, telephone interview or an online interview via Microsoft Teams. For those participants who select a face-to-face interview, these will be conducted at a place and time convenient to them, this may include while they are receiving haemodialysis at one of their routine clinical appointments. Written informed consent will be obtained prior to the commencement of interview. Participants who would like to participate in a telephone or virtual interview, will be asked to scan or take a digital photograph of their signed, dated and initialled informed consent form and send this via email or post to the research team. Interviews will not commence until written informed consent has been obtained. Carers will have the option of a home, telephone or virtual interview via Microsoft teams and the same process will be applied regarding written informed consent. A lone working policy will be implemented for domiciliary interviews and a Covid-19 risk assessment will be adhered to for domiciliary and hospital site interviews.

### Semi-structured interview questions

In accordance with interpretative phenomenological research, an interview guide for use within the semi-structured interviews has been developed from the literature and from iterative guidance from the research team. When developing interview guides, Riessman [32] suggests using five to seven broad areas of interest relating to the research topic. The interview guides, used for both individuals with renal cachexia and their carers in this study contains a flexible format. This allows room to phrase questions spontaneously, to probe, clarify and reflect (Table 2). To ensure the interviewees understand the questions asked by the interviewer, we refer to ‘weight loss’ in the interview schedule instead of referring to renal cachexia. The interviewer is a skilled and experienced researcher and will base all probes on Evan’s et al.’s [1] definition of cachexia to ensure that the holistic experience of living with cachexia is comprehensively captured through the interview questions. Each participant will be asked similar opening questions (‘I am interested to hear about the weight loss you have/your loved one has had; can you tell me about it? How would you describe your/your loved one’s experience of weight loss?). The subsequent interview questions will be largely participant-driven and open as each participant will most probably respond to the questions as per their experiences in an unstructured manner [40]. The subsequent follow up questions are in seven areas of interest on topics that emanated from the literature review, the research team’s guidance along with strategies required to facilitate open communication. Specific clarifying questions will be used to provide more detail on their reflections, experiences and perspectives. Furthermore, in keeping with the Husserlian approach [43], contextualisation questions will be utilized to provide additional focus for these experiences. For example, questions about their thoughts (What are your thoughts about the weight loss you/your loved one have/has had?) and emotions (How does your/your loved one’s weight loss make you feel?) will give crucial insights into their personal experience of weight loss. Other questions will explicitly focus on their perceptions of self (How does your/your loved one’s weight loss affect how you/they see yourself/themselves?) and social perceptions (How does your/your loved one’s weight loss affect your social relationships with your family and friends?). The impact of weight loss will be explored relating to the changes to everyday life (How does your weight loss affect your everyday life?) as well as on the long-term, all-encompassing impact (What does this weight loss mean for you now?). Finally, an imaginative question will be used (Given your experience, what advice would you give to family, friends and staff involved in caring for those with the same condition in the future?) to add a dynamic element to the interview and explore the phenomenon’s stability [44]. To conclude each interview, a closing question will be asked to both sample groups—‘Is there anything else you would like to say or talk about that I haven’t asked you today?’ [45]. By including this question Morse and Field [45] advise that the researcher could gain crucial data that would have otherwise been lost it is also hoped this will help relieve any stress that the research participants may encounter during the interview process [46].

**Table 2:** Interview schedule.

**Opening Questions**	
I am interested to hear about the weight loss you have / your loved one has had; can you tell me about it?	
How would you describe your / your loved one’s experience of weight loss?	
**Area of Interest**	**Follow-up Questions**
Thoughts	What are your thoughts about the weight loss you / your loved one have/has had?
Emotions	How does your / your loved one’s weight loss make you feel?
Perception of self	How does your / your loved one’s weight loss affect how you/they see yourself/themselves?
Social perception	How does your / your loved one’s weight loss affect your social relationships with your family and friends?
Everyday impact	How does your / your loved one’s weight loss affect your everyday life?
Long-term impact	What does this weight loss mean for you now?
Lived expertise	Given your experience, what advice would you give to family, friends and staff involved in caring for those with the same condition in the future?
**Additional Prompts**	
Could you tell me about…,	
How do you feel about…,	
What does it mean…,	
What do you think…,	
Can you tell me a little more about…,	
How do you see…	
**Debriefing Question**	
Is there anything else you would like to say or talk about that I haven’t asked you today?	

### Analysis

Interpretive Phenomenological Analysis (IPA) will be undertaken to explore the participants’ lived experiences [40]. In IPA, the dialogue between the researcher and the participant is described as a double hermeneutic and IPA recognises the importance of constant self‐awareness and reflexivity [47]. IPA is an approach that allows for an exploration of the experience of an event from an individual’s subjective perspective. In IPA, Smith and Osborn [48] explain the process as one in which “the researcher is trying to make sense of the participant trying to make sense of what is happening to them”. The approach seeks to explore how individual participants make sense of ‘a happening’ in their lives and also to understand the meaning behind these experiences [40] making this method appropriate to explore the lived experience of those with ESRD.

Aligned with the ideographic nature of IPA [49] semi-structured interviews will be used to obtain an in-depth understanding of each individual participant’s subjective lived experiences. To enhance reflexivity, throughout the study, from conception to dissemination, the researcher will write notes in a reflective diary to record observations and reflections about the interview experience, in particular non-verbal communication [50], or any other thoughts and comments of potential significance [47, 51]. This data will also be added to verbatim transcripts at the initial stage of analysis, this approach will clearly show the direction of questioning and help provide the necessary context.

The process of IPA will follow the six steps, as outlined by Smith, Flowers and Larkin [49] (Table 3). Firstly, it will involve listening to the recorded interviews, transcribing, and reading the interview transcripts multiple times. Secondly, it will involve noting anything of potential significance in the margins of the transcripts. Thirdly, statements which uncover the lived experience of living with renal cachexia will be clustered and subsequently developed into themes. Fourthly, emerging themes will be recorded and any correlations between them will be noted. As evolving clustering of themes develop, the initial transcripts will be re-checked to ensure any connections are unambiguous. After verifying connections between themes, a table of themes will be created. Fifthly, analysis will continue with the remaining cases. The themes that emerge from the initial transcript will be used as a guide for subsequent interviews. Thus, analysis will be conducted firstly in the initial transcript and then across all cases. All new themes will be checked with prior transcripts and analysis will continue back and forth between the cases to ensure the themes are representative. Finally, in step six, when the analysis is complete a master list of themes will be generated across all cases. This will require clustering themes together into connected groups to form a clear and accurate table of themes. Two members of the research team (CB and JR) will independently code the data and then compare and contrast coding to aid in refining the final themes. NVivo, will be used for data management [52].

**Table 3:** Smith, Flowers, and Larkin’s [53] six analytic steps of IPA.

Step 1	Reading and re-reading the transcript
Step 2	Initial noting
Step 3	Developing emergent themes
Step 4	Searching for connections across emergent themes
Step 5	Moving on to the next case
Step 6	Identifying patterns across cases

### Rigour

The data collection and subsequent analysis will be stringently monitored to ensure rigour within the study. Specific approaches to uphold rigour include digital recording and verbatim transcription of all interviews to provide credibility. A process of confirming meaning by using reflective questioning within the interviews will be used [54]. In addition, the interviewer (CB) will take thorough handwritten field notes of each interview. Those notes will be especially relevant because they help to illuminate the direction of questioning within each interview and provide the necessary context for analysis. The analysis and interpretation of data will be “thick” in that it will include the complexities in the data set [55]. Given that two members (CB and JR) of the team will independently code data and then compare and contrast coding this will also add to the rigour of the study.

### Ethical considerations

Ethical approval for this study was granted by the Office for Research Ethics Committees Northern Ireland (REC Reference: 22/NI/0107). Essential aspects of good practice, including clear and sensitively written participant facing materials, voluntary participation, informed consent, confidentiality, and data protection procedures will be employed as a minimum standard. Written informed consent from participants will be obtained prior to interview and stored securely in a locked office within Queen’s University Belfast (QUB). Confidentiality will be assured as access to raw data will be confined to the research team ensuring the secure storage of voice files and transcripts and voice files deleted after transcription. Data protection procedures will include voice files/transcripts being numbered rather than documenting the participants’ names and the use of pseudonyms in transcripts/quotations. The research team are cognisant that the interviews have the potential to exacerbate emotional distress given the focus on a sensitive topic in a vulnerable population. As such, a distress protocol adapted from Draucker et al. [56] has been developed for use with both individuals with renal cachexia and their carers. Furthermore, the research team are experienced in qualitative research and have designed the study to permit flexibility in questioning during interviews which will allow the emotions of participants to be sensed and questioning to be adapted appropriately. Overall, the recruitment and data collection process has been designed to reduce the burden on participants as much as possible.

### Personal and Public Involvement and Engagement (PPIE)

This protocol has been developed with advice and contribution through engagement with individuals experiencing renal disease. At the outset this research project was aligned with the Health and Social Care Personal and Public Involvement (PPI) policy. Early and ongoing PPIE has been facilitated through our lead representative Mr William Johnston (co-author) from Northern Ireland Kidney Patients Association (NIKPA) to refine the study, ensure clarity and affirm the importance of PPIE. WJ has reviewed participant facing materials, this protocol and will review subsequent publications and the dissemination of research results to the community and professionals. To improve and document the quality, transparency, and consistency of PPIE in this study the Guidance for Reporting Involvement of Patients and the Public-Short Format (GRIPP2-SF) will be followed [57].

## Discussion

The research will provide in-depth understanding into a highly complex phenomenon. As evidenced from the research conducted in renal cachexia, current emphasis is largely placed on the anatomical, physiological and pathological issues central to cachexia in renal disease [7]. The current gap within the literature indicates an urgent need for research to help fully understand the lived experience of individuals who have renal cachexia and their carers. Further research is needed to investigate models of cachexia management in this population, working towards uniform criterion for screening, diagnosis and optimum therapy to update and create relevant clinical guidelines [5, 14–17, 58]. Therefore, the aim of this research in conducting an in-depth phenomenological exploration of the experiences of living with renal cachexia for individuals with ESRD and interrelated experiences of their carers, will contribute significantly to bridging this research gap. This study will help to identify the needs of those with renal cachexia and build an evidence base to support increasing provision in relation to relevant services, such as support groups, psychological therapy and educational programs.

Understanding the lived experience of those with renal cachexia will help to inform better information provision to individuals diagnosed with renal disease, their carers and staff involved in care delivery which both recognises and responds to the needs to this population.

## Conclusions

The proposed research will provide an understanding of the experience of renal cachexia. Through increasing awareness of the impact of renal cachexia we aim to inform multidisciplinary interventions, and the dissemination of this research will add to the ongoing development of holistic care within nephrology. This research will also potentially provide opportunities for future research, for example, understanding the differences and similarities between the lived experience of cachexia in other chronic diseases compared with renal cachexia. In addition, when triangulated with existing studies relating to renal cachexia [5, 8, 14–16], this prospective research may have global impact, informing future practice guidelines specifically on renal cachexia, more generally on renal disease and increasingly the wider understanding of cachexia in chronic diseases.

## Supporting information

S1 File Proforma for consenting participants. (DOCX)
