# Supplementary material for: Exploring the lived experience of renal cachexia for individuals with end-stage renal disease and the interrelated experience of their carers: Study protocol
Source: PLoS One. 2022 Nov 3;17(11):e0277241. doi: 10.1371/journal.pone.0277241 (PMC9632830; doi:10.1371/journal.pone.0277241)
Supplement: S1 File — (DOCX) [file pone.0277241.s001.docx]

**Supplementary document 1**: Proforma for consenting participants

| ***Renal Cachexia Study (NHSCT/QUB): Proforma for participants who have consented to interview***  **To be completed by a member of the clinical care team post receiving confirmation of consent from a member of the QUB research team. **Please tick the relevant box or fill in the information in the box provided.* | |
| --- | --- |
| 1. Participant ID: |  |
| 1. Age: |  |
| 1. Gender (identifies as): | Male Female Other |
| 1. In receipt of haemodialysis: | Yes No |
| 1. Dialysis vintage (years): |  |
| 1. Haemoglobin (g/dl): |  |
| 1. Oedema-free weight loss of at least 5% in 12 months or less and/or a BMI <20.0 kg/m2: | Yes No |
| 1. Experiencing weight loss due to other physical causes, for example: malabsorption (i.e prolonged nausea or vomiting; oesophageal blockage; bowel obstruction; persistent diarrhoea), starvation, primary depression, hyperthyroidism and age-related loss of muscle mass. | Yes No |
| 1. Experiencing fatigue - defined as physical and/or mental weariness resulting from exertion; an inability to continue exercise at the same intensity with a resultant deterioration in performance. | Yes No |
| 1. Experiencing anorexia - limited food intake (i.e. total caloric intake less than 20 kcal/kg body weight/d, <70% of usual food intake) or poor appetite. | Yes No |
| 1. Inflammatory markers (CRP mg/l, pg/ml): |  |
| 1. Serum albumin (g/dl): |  |
| **Thank you for taking the time to complete this proforma.** | |
